# Identification of Fungal Metabolite Gliotoxin as a Potent Inhibitor Against Bacterial *O*-Acetylserine Sulfhydrylase CysK and CysM

**DOI:** 10.3390/ijms26031106

**Published:** 2025-01-27

**Authors:** Azizur Rahman, Katsuhiko Ono, Touya Toyomoto, Kenjiro Hanaoka, Tomohiro Sawa

**Affiliations:** 1 Department of Microbiology, Graduate School of Medical Sciences, Kumamoto University, 1-1-1 Honjo, Chuo-ku, Kumamoto 860-8556, Japan; azizur.rahman.789@gmail.com (A.R.); onokat@kumamoto-u.ac.jp (K.O.); kuma.toyomoto@gmail.com (T.T.); 2 Graduate School of Pharmaceutical Sciences, Keio University, 1-5-30 Shibakoen, Minato-ku, Tokyo 105-8512, Japan; khanaoka@keio.jp

**Keywords:** *O*-acetylserine sulfhydrylase, gliotoxin, cysteine, antibacterial, antimicrobial resistance

## Abstract

Cysteine is an essential amino acid for sustaining life, including protein synthesis, and serves as a precursor for antioxidant glutathione. Pathogenic bacteria synthesize cysteine via a two-step enzymatic process using serine as the starting material. The first step is catalyzed by serine acetyltransferase, also known as CysE, and the second by *O*-acetylserine sulfhydrylase (OASS), referred to as CysK or CysM. This cysteine biosynthetic pathway in bacteria differs significantly from that in mammals, making it an attractive target for the development of novel antibacterial agents. In this study, we aimed to identify OASS inhibitors. To achieve this, a high-throughput screening system was developed to analyze compounds capable of inhibiting CysK/CysM activity. Screening 168,640 compounds from a chemical library revealed that gliotoxin, a fungal metabolite, strongly inhibits both CysK and CysM. Furthermore, gliotoxin significantly suppressed the growth of *Salmonella enterica* serovar Typhimurium, a Gram-negative bacterium, under cystine-deficient conditions. Gliotoxin possesses a unique disulfide structure classified as epipolythiodioxopiperazine. To date, no studies have reported OASS inhibition by compounds with this structural motif, highlighting its potential for future structural optimization. The screening system developed in this study is expected to accelerate the discovery of functional CysK/CysM inhibitors, providing a foundation for novel antibacterial strategies.

## 1. Introduction

In recent years, the emergence of drug-resistant bacteria with resistance against antibiotics traditionally considered as last-resort treatments, such as vancomycin and carbapenems, has become a pressing global issue [1,2,3]. Addressing this challenge is of critical importance, as the emergence of resistant strains is associated with poor clinical outcomes, especially in vulnerable populations such as the elderly and infants, and contributes to increased healthcare costs [1,2,3]. Combating antimicrobial resistance requires a multifaceted approach, including the appropriate use of antibiotics, strengthening surveillance systems, and the development of novel therapeutic strategies. Recent studies have reported that reactive oxygen species (ROS) generated within bacterial cells during antibiotic treatment synergistically enhance the bactericidal effects of these drugs [4,5]. This phenomenon has been observed with a wide range of antibiotics, including β-lactams, aminoglycosides, macrolides, and tetracyclines, suggesting a global mechanism of antimicrobial action enhancement [4,5]. Therefore, selectively weakening bacterial oxidative stress resistance is expected to enhance the bactericidal effects of antibiotics and host immune responses [6,7].

**Figure 1:**
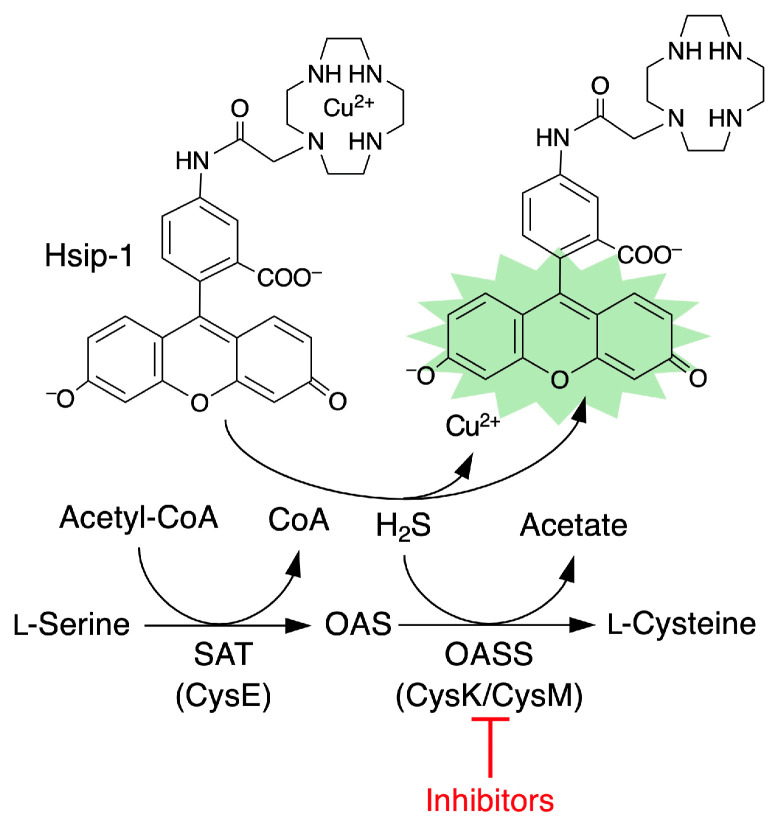
Cysteine biosynthesis pathway in bacteria. CoA, coenzyme A; OAS, *O*-acetylserine, OASS, *O*-acetylserine sulfhydrylase; SAT, serine acetyltransferase. In the first step, SAT catalyzes the acetylation of serine to form OAS. In the second step, OASS catalyzes cysteine formation from OAS with the use of hydrogen sulfide (H_2_S) as a co-substrate. In this study, OASS inhibitor screening was carried out by determining the consumption of H_2_S during the second step enzyme reaction. A highly sensitive H_2_S probe HSip-1 was used to monitor H_2_S consumption.

Cysteine is an essential amino acid required for protein synthesis and is also a precursor for the synthesis of glutathione, a key intracellular antioxidant [8,9]. Most pathogenic bacteria synthesize cysteine via a two-step enzymatic reaction, starting from serine (Figure 1) [10]. The first step is catalyzed by serine acetyltransferase (SAT, also known as CysE), and the second by *O*-acetylserine (OAS) sulfhydrylase (OASS, also referred to as CysK or CysM). CysK and CysM are isoenzymes and catalyze the same enzyme reaction for the conversion of OAS to cysteine with the use of hydrogen sulfide (H_2_S) as a co-substrate. CysM also utilizes thiosulfate, instead of hydrogen sulfide, as a co-substrate for cysteine biosynthesis [11]. These bacterial cysteine biosynthesis pathways differ significantly from those in mammals, making them attractive targets for the development of novel antibiotics [10,12,13,14]. Furthermore, recent studies have revealed that cysteine is metabolized into more chemically reactive derivatives, cysteine persulfide and polysulfides, which exhibit diverse biological activities [15,16,17,18]. Interestingly, bacterial-derived cysteine persulfide and polysulfides have been shown to react with β-lactam antibiotics, directly inactivating them and contributing to intrinsic resistance mechanisms [19].

In our previous research, we identified specific inhibitors of CysE, demonstrating that alkyl gallates, particularly octyl gallate, strongly inhibit CysE activity and significantly reduce bacterial levels of cysteine and glutathione [12]. Moreover, octyl gallate was found to enhance bacterial susceptibility to carbapenem antibiotics [12]. In this study, we focused on identifying inhibitors targeting the second step of bacterial cysteine biosynthesis, catalyzed by CysK/CysM. To achieve this, we established a high-throughput screening system capable of identifying compounds that inhibit CysK/CysM. Screening a library of approximately 170,000 compounds, we identified gliotoxin, a fungal metabolite, as a potent inhibitor of both CysK and CysM. Gliotoxin also exhibited strong growth-inhibitory effects against *Salmonella* enterica serovar Typhimurium (*S.* Typhimurium), a Gram-negative bacterium, under cystine-deficient conditions. Gliotoxin is a highly toxic mycotoxin produced by a number of *Gliocladium*, *Aspergillus*, and *Penicillium* species [20]. It has been reported to be primarily immunosuppressive [21], but also to have antibacterial and antiviral activities as well as apoptotic, cytotoxic, and genotoxic effects [22]. Gliotoxin possesses a unique disulfide bond structure known as an epipolythiodioxopiperazine [23], a feature not previously associated with OASS inhibition [14]. These findings suggest that gliotoxin and related compounds hold promise for future structural optimization as novel antimicrobial agents. The high-throughput screening system developed in this study is anticipated to accelerate the discovery of functional CysK/CysM inhibitors, contributing to the advancement of antimicrobial therapy against resistant bacteria.

## 2. Results

### 2.1. Development of an OASS Inhibitor Screening System

In this study, we established a functional OASS inhibitor discovery system by combining enzyme-based high-throughput screening with bioassays that evaluate bacterial growth inhibition.

Initially, we developed a high-throughput screening system using recombinant CysK and CysM enzymes to identify inhibitors. CysK and CysM catalyze the reaction between OAS and H_2_S to produce cysteine (Figure 1). We monitored the consumption of H₂S during this reaction using HSip-1, a fluorescent probe reactive to H_2_S [24,25].

**Figure 2:**
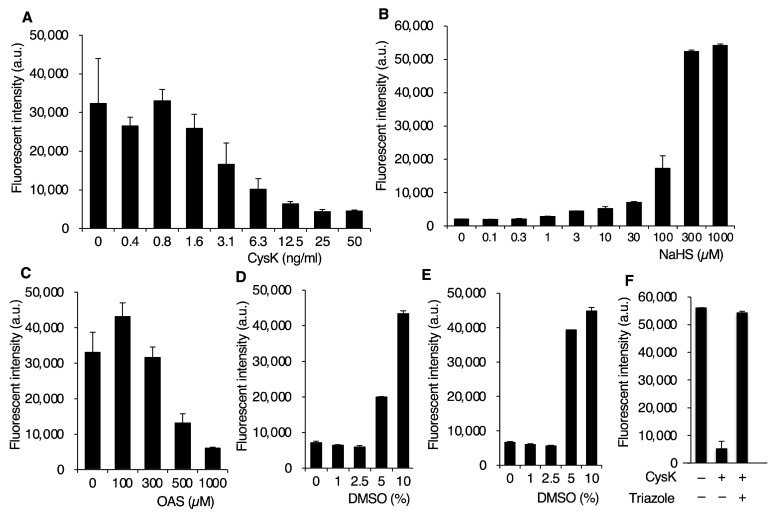
Optimization of enzyme-based OASS inhibitor screening system. HSip-1 derived fluorescent intensities were measured as functions of concentrations of (**A**) CysK, (**B**) NaHS, (**C**) OAS, and (**D**,**E**) DMSO. Reaction mixtures in (**A**) consist of 100 μM OAS, 100 μM NaHS, 1 μM HSip-1 with indicated concentrations of CysK. In (**B**), HSip-1 (1 μM) was incubated in the presence of indicated concentrations of NaHS. (**C**) Various concentrations of OAS were incubated in the presence of 300 μM NaHS and 25 ng/mL CysK. Effects of DMSO on OASS reactions catalyzed by CysK (**D**) and CysM (**E**) were determined. Reaction mixtures consist of 1 mM OAS, 300 μM NaHS, and 25 ng/mL CysK (**D**) or CysM (**E**) in the presence of indicated concentrations of DMSO. (**F**) Inhibition of CysK reaction by 1,2,4-triazole. HSip-1 derived fluorescent intensities were measured for reaction mixtures containing 300 μM NaHS and 1 mM OAS with (+) or without (–) CysK (25 ng/ml) and 1,2,4-triazole (Triazole). CysK-dependent consumption of H_2_S was completely cancelled in the presence of 500 μM 1,2,4-triazole. Data are means ± SD (*n* = 3).

Fluorescence intensity derived from HSip-1 decreased in a concentration-dependent manner with CysK, plateauing at concentrations above 25 ng/mL (Figure 2A). Similar results were obtained with CysM (Supplementary Figure S1). The fluorescence intensity of HSip-1 increased with the addition of NaHS, reaching a plateau at 300 μM (Figure 2B). The other substrate, OAS, significantly suppressed fluorescence intensity at a concentration of 1 mM (Figure 2C). Based on these results, we set the reaction conditions as follows: enzyme concentration at 25 ng/mL, OAS concentration at 1 mM, and NaHS concentration at 300 μM.

We next examined the impact of dimethyl sulfoxide (DMSO) concentration, as samples from compound libraries are dissolved in DMSO. CysK and CysM enzymatic activities were unaffected by DMSO concentrations up to 2.5% (Figure 2D,E). However, at concentrations exceeding 5%, CysK activity was inhibited by approximately 50%, and complete inhibition occurred at 10% DMSO (Figure 2D). Similarly, CysM activity was almost entirely inhibited at DMSO concentrations exceeding 5% (Figure 2E). Consequently, we set the DMSO concentration at 2.5%. Finally, to validate the functionality of this screening system, we tested 1,2,4-triazole, a known inhibitor of CysK [26]. In the presence of 1,2,4-triazole, fluorescence intensity derived from HSip-1 increased, indicating enzymatic inhibition (Figure 2F). Using a 384-well plate and an automated dispenser, our system enabled the screening of approximately 10,000 compounds per day.

### 2.2. High-Throughput Screening of OASS Inhibitors

We conducted a first-round screening of 168,640 compounds at a fixed concentration of 25 μM, using a 50% inhibition threshold as the cutoff for hit identification. A heatmap representing enzyme activity (0–100%) was generated for each 384-well plate (Supplementary Figure S2). For example, plate A displayed strong inhibition in well E-12, and plate B exhibited inhibitory activity in multiple wells.

**Figure 3:**
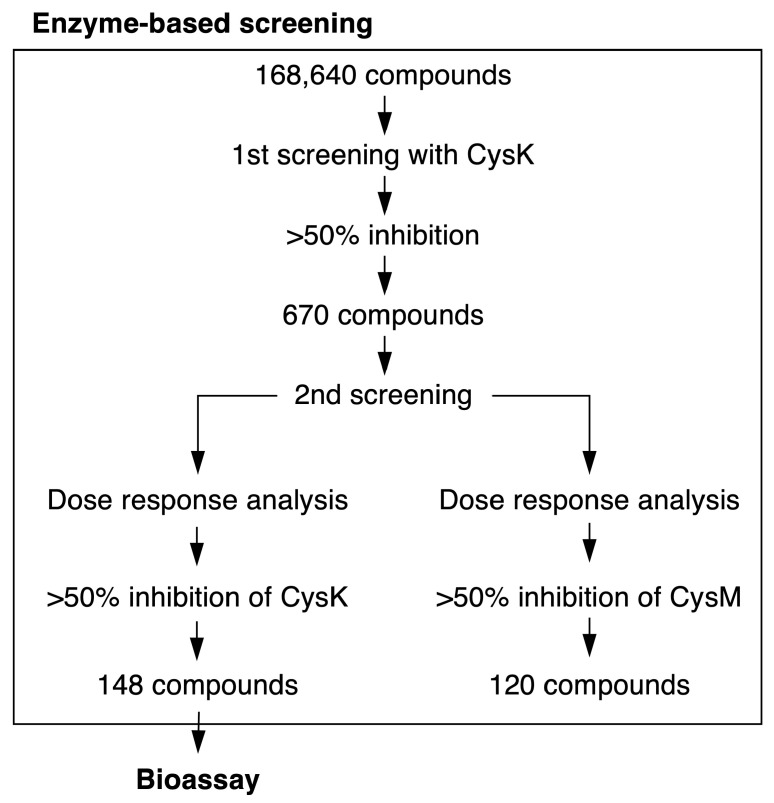
Summary of enzyme-based screening of OASS inhibitors. In total, 168,640 compounds were subjected to the first round of enzyme-based screening of OASS inhibitors. The 148 compounds selected based on the second round of screening were subjected to a bioassay.

This primary screening identified 670 compounds that inhibited CysK activity by over 50% (Figure 3, Supplementary Figure S3). We further analyzed the reproducibility and dose dependence of the inhibitory effects of these hits. Among them, 148 compounds inhibited CysK by over 50%, while 120 compounds showed similar inhibition for CysM (Figure 3). A total of 74 compounds inhibited both enzymes. The 148 CysK inhibitors were subsequently evaluated in a bacterial growth inhibition assay.

### 2.3. Development of a Bioassay for OASS Inhibitors

**Figure 4:**
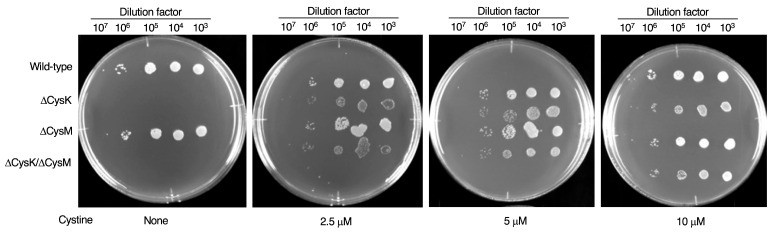
Growth suppression of CysK-deficient *E. coli* on M9 agar plates. *E. coli* wild-type strain and/or *E. coli* mutants lacking CysK, CysM, or both CysK and CysM were plated on M9 agar plates supplemented with different concentrations of cystine (0~10 μM), and cultured for 24 h. No visible colonies were observed for CysK and CysK/CysM-deficient bacteria on M9 agar plates without cystine supplementation.

As shown in Figure 4, wild-type *Escherichia coli* (*E. coli)* grew on minimal M9 agar medium containing glucose as the sole carbon source. However, *E. coli* lacking CysK did not grow on this medium, whereas the CysM-deficient strain exhibited growth comparable to the wild type (Figure 4). Adding cystine to the M9 agar restored the growth of the CysK-deficient strain in a dose-dependent manner, suggesting that this assay could identify compounds with strong OASS inhibitory activity.

**Figure 5:**
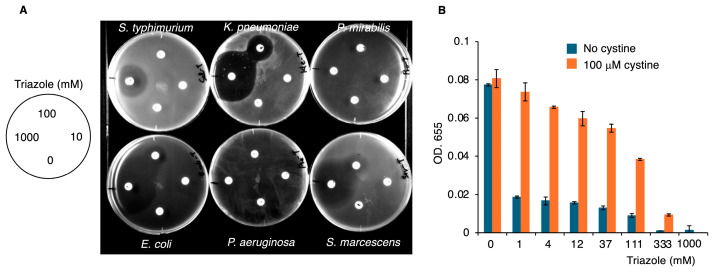
Growth-inhibitory effects of 1,2,4-triazole for Gram-negative bacteria. (**A**) Paper discs soaked in different concentrations of 1,2,4-triazole (0, 10, 100, and 1000 mM) were placed on M9 agar plates pre-coated with bacteria. (**B**) *K. pneumoniae* were cultured in M9 medium with or without 100 μM cystine in the presence of indicated concentrations of 1,2,4-triazole for 24 h.

Using 1,2,4-triazole, we evaluated the assay’s effectiveness. When various Gram-negative bacteria were cultured on M9 agar-containing paper discs soaked with different concentrations of 1,2,4-triazole, clear inhibition zones were observed for *S.* Typhimurium, *Klebsiella pneumoniae* (*K. pneumoniae)*, *E. coli*, and *Serratia marcescens* (Figure 5). In liquid M9 medium, *K. pneumoniae* growth was significantly inhibited by 1,2,4-triazole, and 75% inhibition of bacterial growth was determined for 1,2,4-triazole at 1 mM (Figure 5B). This growth inhibition by 1,2,4-triazole was reversed upon cystine addition, however (Figure 5B). These results indicate the utility of M9 agar-based and liquid-medium-based bioassays for identifying OASS inhibitors.

### 2.4. Induction of Oxidative Stress Sensitivity by CysK Deletion

**Figure 6:**
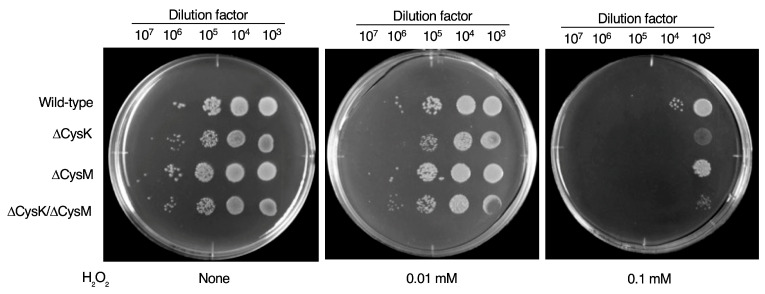
Enhanced vulnerability for CysK-deficient *E. coli* against hydrogen peroxide-induced oxidative stress. *E. coli* wild-type strain and/or *E. coli* mutants lacking CysK, CysM, or both CysK and CysM were plated on M9 agar plates containing 2.5 μM cystine supplemented with different concentrations of hydrogen peroxide (H_2_O_2_) (0~0.1 mM) and cultured for 24 h.

We hypothesized that the restriction of endogenous cysteine supply due to CysK deletion reduces glutathione, an antioxidant peptide, leading to increased sensitivity to oxidative stress. To test this, we compared the oxidative stress tolerance of *E. coli* strains lacking CysK with that of the wild type. In M9 agar medium supplemented with 2.5% cystine to support minimal growth of CysK-deficient *E. coli*, the addition of hydrogen peroxide inhibited bacterial growth in a concentration-dependent manner (Figure 6). The growth inhibition caused by hydrogen peroxide was significantly more pronounced in the CysK-deficient strain than in the wild type. These findings suggest that CysK contributes to oxidative stress tolerance in *E. coli*.

### 2.5. Bioassay of CysK Inhibitors

**Figure 7:**
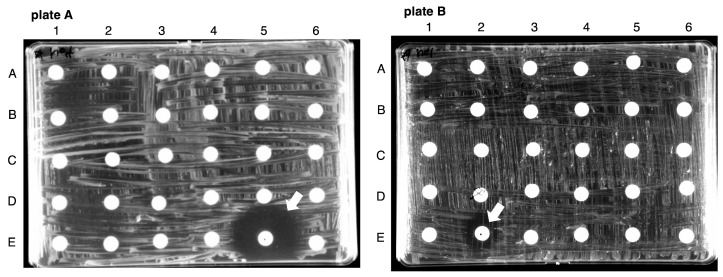
Bioassay for OASS inhibitors. Paper discs soaked in 400 μM compound were placed on M9 agar plates pre-coated with *Salmonella* Typhimurium and cultured for 24 h. Arrows indicate a clear zone of inhibition on agar plates (E5 in plate A, E2 in plate B). A total of 148 compounds were subjected to a bioassay (plate A, B, and C). Data for plate C to plate E are shown in Supplementary Figure S4.

The 148 compounds identified as CysK inhibitors in the high-throughput screening were further evaluated for bacterial growth inhibition using bioassays. *S.* Typhimurium was spread on M9 agar, and paper discs impregnated with 400 μM of each compound were placed on the agar for a 24 h incubation. Among the tested compounds, distinct inhibition zones were observed around the E-5 disc on plate A and the E-2 disc on plate B (Figure 7). No inhibition zones were detected on other plates (Supplementary Figure S4).

**Figure 8:**
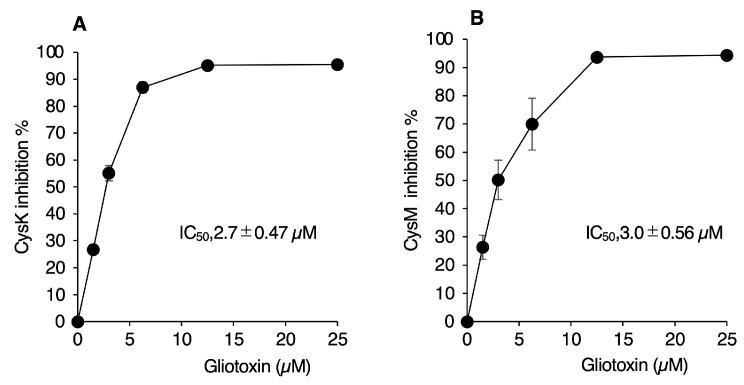
Inhibition of recombinant CysK (**A**) and CysM (**B**) by gliotoxin. Enzyme reactions were monitored by measuring the consumption of H_2_S with the use of HSip-1. Enzyme reactions contained 25 ng/mL enzyme, 300 μM NaHS, 1 mM OAS, and indicated concentrations of gliotoxin. Data are means ± SD (*n* = 3). IC_50_ values for gliotoxin against CysK and CysM are shown in the figure.

The compound on disc E-5 of plate A was identified as gliotoxin, a fungal metabolite. Gliotoxin strongly inhibited recombinant CysK and CysM activities, with an estimated IC_50_ of approximately 3 μM based on H_2_S consumption (Figure 8). Gliotoxin possesses a disulfide bond in its structure (see below) which may react with thiol-containing molecules including H_2_S. In order to confirm whether gliotoxin interferes in an OASS inhibitor assay via direct interaction with H_2_S, fluorescent intensity derived from HSip-1 was monitored in the presence of 300 μM NaHS with varying concentrations of gliotoxin. As shown in Supplementary Figure S5, fluorescent intensity was only slightly decreased even in the presence of 25 μM gliotoxin. This indicates that the current OASS inhibitor assay can be performed in the presence of gliotoxin. To further verify that gliotoxin indeed inhibits OASS reaction, cysteine production from OASS reactions was determined in the presence or the absence of gliotoxin. As shown in Supplementary Figure S6, gliotoxin strongly inhibited cysteine production from CysK and CysM reactions to a similar extent to that determined by the HSip-1-based assay. These data suggest that gliotoxin can act as a potent inhibitor against CysK/CysM-catalyzed OASS reactions. Although gliotoxin has known antibacterial activity, this is the first report of its inhibitory effects on CysK and CysM.

The compound on disc E-2 of plate B was identified as 2-oxo-1,3-dithiole-4,5-dicarboxamide, a synthetic compound (Supplementary Figure S7). This compound inhibited the growth of *K. pneumoniae* in M9 liquid medium in a concentration-dependent manner, but its inhibitory effects were not reversed by cystine addition (Supplementary Figure S7). These results suggest that 2-oxo-1,3-dithiole-4,5-dicarboxamide inhibits bacterial growth through a mechanism distinct from OASS inhibition.

### 2.6. Antibacterial Activity of Gliotoxin

Gliotoxin possesses a disulfide bond in its structure, which has been reported to play a key role in its various biological activities [23,27]. To investigate the impact of this disulfide bond on bacterial growth, we examined bis(methylthio)gliotoxin, a derivative in which the disulfide bond was reduced and the resulting thiol groups were methylated. For this experiment, gliotoxin was purchased separately rather than using a chemical library.

**Figure 9:**
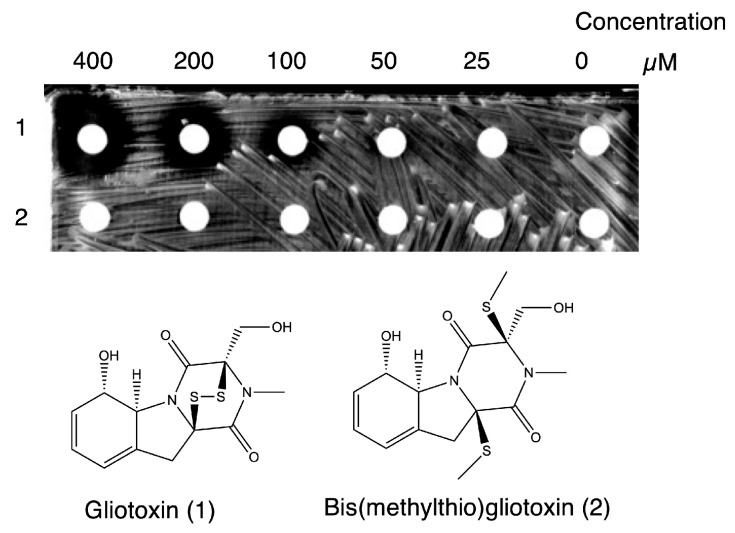
Dose-dependent inhibition by gliotoxin of *S.* Typhimurium cultured on an M9 agar plate. Paper discs soaked on M9 medium containing different concentrations of (1) gliotoxin and its derivative (2) bis(methylthio)gliotoxin (0–400 μM) were placed on an M9 agar plate and cultured for 24 h.

**Figure 10:**
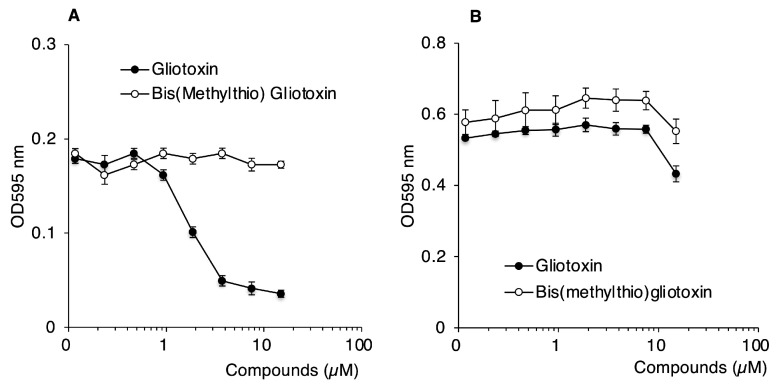
Effects of gliotoxin and bis(methylthio)gliotoxin on the growth of *S*. Typhimurium. *S*. Typhimurium were cultured in M9 medium (**A**) and in LB medium (**B**) containing indicated concentrations of the reagents for 24 h. Bacterial growth was determined by measuring turbidity. Data are means ± SD (*n* = 3).

As shown in Figure 9, gliotoxin inhibited the growth of *S*. Typhimurium in a concentration-dependent manner. This result confirmed that gliotoxin itself exhibits bacterial growth inhibition. In contrast, bis(methylthio)gliotoxin showed no effect on bacterial growth, even at the maximum concentration of 400 µM. Similar results were obtained using an M9 liquid medium (Figure 10). Gliotoxin inhibited the growth of *S*. Typhimurium with an IC_50_ of approximately 2 µM. Conversely, bis(methylthio)gliotoxin showed no growth-inhibitory activity. When LB medium was used, the growth-inhibitory effect of gliotoxin was markedly diminished, showing only a slight effect at 20 µM. A previous study reported that LB medium contains cystine in a range of 90 μM or higher [28]. It is thus considered that cystine contained in LB medium cancels out the effects of gliotoxin. At this concentration, gliotoxin may suppress bacterial growth through a mechanism distinct from OASS inhibition.

## 3. Discussion

### 3.1. Exploration of Bacterial Cysteine Biosynthesis Pathway as an Antimicrobial Target

The bacterial cysteine biosynthesis pathway has garnered significant attention as a promising target for antimicrobial drug development [10,11,12,13,14]. Recently, we identified octyl gallate as an inhibitor of serine acetyltransferase (SAT, CysE) and demonstrated its antibacterial activity against Gram-negative bacteria. Additionally, we reported its potential to enhance the susceptibility of drug-resistant bacteria to existing antibiotics [12]. Compounds that enhance antibiotic efficacy, even without intrinsic antimicrobial activity, are referred to as “antibiotic adjuvants”. These adjuvants are being explored as innovative solutions to restore drug susceptibility in resistant bacterial strains, attracting considerable research interest [29,30,31]. Compounds that inhibit bacterial cysteine biosynthesis hold potential as antibiotic adjuvants.

In this study, we focused on identifying inhibitors of OASS, the enzyme catalyzing the second step of bacterial cysteine biosynthesis. We developed a high-throughput screening system using recombinant enzymes and employed the fluorescent probe HSip-1 to quantify the consumption of H_2_S, a substrate of OASS, with high sensitivity [24,25]. This approach enabled us to minimize reaction volumes to 20 µL and required as little as 12.5 pmol of each compound (25 µM in 0.5 µL). By utilizing an automated liquid handling system, we achieved a throughput of 10,000 compounds per day.

### 3.2. OASS Inhibitor Screening

We screened 168,640 compounds provided by the University of Tokyo Drug Discovery Initiative (DDI) and identified 670 compounds that inhibited CysK activity by over 50% (Figure 3). Further analysis revealed 148 compounds selectively inhibiting CysK and 120 compounds targeting CysM (Figure 3). Notably, 74 compounds inhibited both CysK and CysM, likely targeting structural features shared by these enzymes, which exhibit 43% sequence similarity and use pyridoxal 5′-phosphate as a cofactor [32,33,34]. Conversely, 74 compounds selectively inhibited CysK, while 46 specifically targeted CysM, suggesting these compounds act on distinct structural or functional sites unique to each enzyme.

Inhibitors of CysM have been reported to suppress biofilm formation by *Streptococcus suis* [35] and to inhibit the growth of dormant *Mycobacterium tuberculosis* [36]. Expanding the scope of CysM inhibitor screening could uncover additional candidates that were not identified in the present study.

### 3.3. Discovery of Gliotoxin as a Potent OASS Inhibitor

We identified gliotoxin, a fungal toxin, as a strong inhibitor of both CysK and CysM (Figure 8), with potent growth-inhibitory effects against *S*. Typhimurium (Figure 9 and Figure 10). Structural analyses indicated that gliotoxin’s disulfide bond is critical for its antibacterial activity. The disulfide bond of gliotoxin is reactive against thiol-containing molecules, such as glutathione (GSH), cysteine, and protein sulfhydryl groups. Previous studies reported that the disulfide bond of gliotoxin could react with GSH, resulting in the generation of the dithiol form of gliotoxin [37,38], or the formation of GSH–gliotoxin conjugates [39]. The electrophilic nature of gliotoxin may, thus, decrease intrabacterial levels of free thiols, which contributes to the observed antibacterial effects. Importantly, gliotoxin-mediated inhibition of cysteine biosynthesis may cooperatively decrease thiol-based antioxidant capacity in bacteria, contributing to potential bacterial killing.

Gliotoxin represents a novel class of OASS inhibitors, distinct from previously reported compounds such as polypeptides [40], cyclopropanes [41,42,43,44,45], thiazolidinones [46], urea derivatives [35,47], fluoroalanine [48], and triazoles [49]. Gliotoxin’s unique epipolythiodioxopiperazine (ETP) structure, shared by other fungal toxins such as sirodesmin and hyalodendrin, provides a promising scaffold for further development [22,23].

### 3.4. Cysteine Uptake Systems as Potential Targets

The antibacterial activity of 1,2,4-triazole and gliotoxin was significantly diminished in cystine-rich media, as bacteria efficiently import cysteine/cystine from their environment. This process is mediated by cystine transporters such as YdjN and the FliY-YecSC system [50,51,52]. Therefore, identifying inhibitors of these transporters is a crucial future direction for enhancing the efficacy of OASS inhibitors.

## 4. Materials and Methods

### 4.1. Materials

The following reagents and chemicals were used in this study. NaHS, cysteine, cystine, Na_2_HPO_4_, NaH_2_PO_4_, NH_4_Cl, CaCl_2_, glucose, DMSO, formic acid, and acetonitrile were purchased from Fujifilm Wako Pure Chemical Corporation, Ltd. (Osaka, Japan). NaCl and MgSO_4_ were from Nacalai Tesque (Kyoto, Japan). Peptone was obtained from Nihon Seiyaku (Izumisano, Japan). Yeast extract was from the Oriental Yeast Company, Ltd. (Tokyo, Japan). Casamino acids were purchased from Becton, Dickinson and Company (Franklin lakes, NJ, USA), and agar was from Nissui Pharmaceutical Co., Ltd. (Tokyo, Japan). OAS and 1,2,4-triazole were from Tokyo Chemical Industry Company, Ltd. (Tokyo, Japan). Gliotoxin, bis(methylthio)gliotoxin, and Tween-20 were obtained from Sigma Aldrich (St. Louis, MO, USA). 2-Oxo-1,3-dithiole-4,5-dicarboxamide was from Labotest (Halsbrücke, Germany). β-(4-hydroxyphenyl)ethyl iodoacetamide (HPE-IAM) was purchased from Molecular Biosciences (Boulder, CO, USA). HSip-1 was synthesized according to the method described in previous work [20].

### 4.2. Bacterial Strains and Culture Conditions

*E. coli* BW25113 (wild type) and single-gene disruption mutants (*cysK* and *cysM*) were obtained from the National Institute of Genetics (Shizuoka, Japan). The *cysK*/*cysM* double mutant was constructed using P1 phage-mediated transduction to introduce the *cysM* mutation into the *cysK* mutant. Other bacterial strains used in this study, including *S*. Typhimurium, *Klebsiella pneumoniae*, *Proteus mirabilis*, *Pseudomonas aeruginosa*, and *Serratia marcescens*, were from our laboratory stock. Bacteria were grown in LB medium (1% NaCl, 1% peptone, 0.5% yeast extract) or M9 minimal medium (33 mM Na_2_HPO_4_, 22 mM KH_2_PO_4_, 8.6 mM NaCl, 9.4 mM NH_4_Cl, 0.1 mM CaCl_2_, 1 mM MgSO_4_) supplemented with 0.2% glucose and/or 0.2% casamino acids as required. Agar plates containing 1.5% agar were also used for bacterial cultivation.

### 4.3. Recombinant Enzymes

Recombinant CysK and CysM enzymes from *S.* Typhimurium were purified by the same protocol as reported previously [53].

### 4.4. High-Throughput Screening System for CysK Inhibitors

To optimize the conditions for high-throughput screening, reaction mixtures were prepared containing various concentrations of CysK, OAS, and NaHS in 100 mM sodium phosphate buffer (NaPB) pH 7.4 supplemented with 0.005% Tween-20. The reaction mixtures (20 µL) were dispensed into 384-well plates (black, non-binding, flat-bottom, #784900; Greiner Bio-One, Kremsmünster, Austria) and incubated at room temperature for 1 h. As a positive control for CysK inhibition, 1,2,4-triazole was used. Subsequently, HSip-1 (1 µM) was added to the plates, and incubation was continued at room temperature for 3 h. The fluorescence intensity of HSip-1 (Cyclen-AF-Cu^2^⁺ form) was measured by using a fluorescence microplate reader (excitation at 485 nm; emission at 520 nm; infinite F200 PRO, TECAN, Männedorf, Switzerland), and CysK activity was determined based on the fluorescence intensity.

For inhibitor screening, a chemical library containing 168,640 compounds was provided by the Drug Discovery Initiative (DDI) at The University of Tokyo (Tokyo, Japan). The first-round CysK enzyme inhibition assay was performed as follows: First, 10 µL of CysK solution (0.1 µg/mL recombinant CysK, 2 mM OAS, 0.005% Tween-20, and 100 mM NaPB pH 7.4) was dispensed into 384-well plates containing 0.25 µL of library compounds (final concentration: 25 µM) using a Multidrop Combi dispenser (Thermo Fisher Scientific, Waltham, MA, USA). The plates were incubated at room temperature for 1 h, after which 10 µL of NaHS (600 µM) was added to each well. After an additional 1 h incubation at room temperature, 1 µL of HSip-1 (20 µM) was added to the plates, followed by a 3 h incubation at room temperature.

For controls, the reaction mixture treated with DMSO was obtained as the positive control, while the reaction mixture without enzyme was used as the negative control. The fluorescence intensity of HSip-1 (Cyclen-AF-Cu^2^⁺ form) was measured using a fluorescence microplate reader (excitation at 485 nm; emission at 520 nm; PHERAstar Plus, BMG Labtech) according to the manufacturer’s instructions. Compounds exhibiting >50% CysK inhibition (670 compounds) were further analyzed for dose dependence (1.5, 3.125, 6.25, 12.5, and 25 µM) and reproducibility was tested using the same protocol as the first screening.

### 4.5. Bioassay for CysK/CysM Inhibitors

The 148 compounds that exhibited reproducibility and strong inhibition of CysK were further evaluated for their antimicrobial activity. A fresh colony of *S*. Typhimurium was streaked onto M9 agar medium supplemented with 0.2% glucose (M9 + Glc agar). Paper discs (Advantech) were soaked with solutions of the 148 compounds (400 µM each) and placed onto the M9 + Glc agar plates pre-streaked with *S*. Typhimurium. The plates were incubated at 37 °C for 18 h, after which the diameters of the inhibition circle were measured to assess antibacterial activity.

### 4.6. Growth Assay of E. coli CysK, CysM, and CysK/CysM Mutants

*E. coli* wild type and its mutants were cultured overnight at 37 °C in LB medium. The overnight cultures (CFU/mL ≈ 10^9^) were serially diluted (10^3^- to 10^7^-fold) in saline and kept on ice until use. Diluted bacterial suspensions (10 µL each) were spotted onto M9 + Glc agar plates supplemented with various concentrations of cystine (final concentration: 0 to 10 µM). After incubation at 37 °C for 24 h, bacterial growth was assessed by counting the number of colonies formed.

### 4.7. Quantification of Cysteine by LC-MS/MS

To quantify the production of cysteine from OASS reaction, we followed the method as previously described [19]. Briefly, reaction mixtures containing 25 ng/mL recombinant CysK or CysM, 1 mM OAS, 300 µM NaHS, and 50 mM NaPB pH 7.4 with or without 25 µM gliotoxin were incubated at 37 °C for 5 min. To stop the enzyme reaction and derivatize for cysteine, we added 50% methanol containing 2.5 mM HPE-IAM and then incubated the mixture at 37 °C for 15 min. After that, the reaction mixture was diluted 10 times by using 0.1% formic acid. HPE-derivatized cysteine was quantitated by means of LCMS/MS according to the protocol.

### 4.8. Antibacterial Assay

The antibacterial activities of gliotoxin and bis(methylthio)gliotoxin were measured by the serial dilution method. An overnight culture of *S*. Typhimurium was diluted 1000-fold into fresh LB or M9 + Glc media. Bacterial suspensions were plated into a 96-well flat-bottom microplate (0.1 mL/well) and were treated with various concentrations (0 to 15 µM) of gliotoxin or bis(methylthio)gliotoxin. After inoculation at 37 °C for 24 h, bacterial growth was assessed by measuring the optical density at 595 nm with a microplate reader (Bio-Rad, Hercules, CA, USA).

## 5. Conclusions

In summary, we developed a high-throughput screening system for OASS inhibitors using recombinant enzymes and employed the fluorescent probe HSip-1 to quantify hydrogen sulfide consumption, a substrate of OASS, with high sensitivity. This approach enabled us to complete the screening of 10,000 compounds per day. Combined with bioassay, a fungal metabolite gliotoxin was identified as a potent inhibitor against both CysK and CysM. Analysis using a gliotoxin analogue, bis(methylthio)gliotoxin, revealed that the unique disulfide structure ETP may be essential for gliotoxin’s action against OASS. Future studies should elucidate gliotoxin’s mechanism of action and target sites within OASS. Additionally, investigating the OASS inhibitory potential of other ETP-containing compounds could lead to the discovery of more potent inhibitors.

## Data Availability

Data are contained within the article.

## Supplementary Materials

The following supporting information can be downloaded at https://www.mdpi.com/article/10.3390/ijms26031106/s1.

## References

[1] Murray C.J.L., Ikuta K.S., Sharara F., Swetschinski L., Aguilar G.R., Gray A., Han C., Bisignano C., Rao P., Wool E. (2022). Global Burden of Bacterial Antimicrobial Resistance in 2019: A Systematic Analysis. Lancet.

[2] Jean S.-S., Harnod D., Hsueh P.-R. (2022). Global Threat of Carbapenem-Resistant Gram-Negative Bacteria. Front. Cell. Infect. Microbiol..

[3] Ma J., Song X., Li M., Yu Z., Cheng W., Yu Z., Zhang W., Zhang Y., Shen A., Sun H. (2023). Global Spread of Carbapenem-Resistant Enterobacteriaceae: Epidemiological Features, Resistance Mechanisms, Detection and Therapy. Microbiol. Res..

[4] Kohanski M.A., Dwyer D.J., Hayete B., Lawrence C.A., Collins J.J. (2007). A Common Mechanism of Cellular Death Induced by Bactericidal Antibiotics. Cell.

[5] Dwyer D.J., Belenky P.A., Yang J.H., MacDonald I.C., Martell J.D., Takahashi N., Chan C.T.Y., Lobritz M.A., Braff D., Schwarz E.G. (2014). Antibiotics Induce Redox-Related Physiological Alterations as Part of Their Lethality. Proc. Natl. Acad. Sci. USA.

[6] Xiao G., Li J., Sun Z. (2023). The Combination of Antibiotic and Non-Antibiotic Compounds Improves Antibiotic Efficacy against Multidrug-Resistant Bacteria. Int. J. Mol. Sci..

[7] Alfei S., Schito G.C., Schito A.M., Zuccari G. (2024). Reactive Oxygen Species (ROS)-Mediated Antibacterial Oxidative Therapies: Available Methods to Generate ROS and a Novel Option Proposal. Int. J. Mol. Sci..

[8] Leustek T., Martin M.N., Bick J.-A., Davies J.P. (2000). Pathways and regulation of sulfur metabolism revealed through molecular and genetic studies. Annu. Rev. Plant Biol..

[9] Kessler D. (2006). Enzymatic Activation of Sulfur for Incorporation into Biomolecules in Prokaryotes. FEMS Microbiol. Rev..

[10] Verma D., Gupta V. (2021). New Insights into the Structure and Function of an Emerging Drug Target CysE. 3 Biotech.

[11] Tai C.H., Nalabolu S.R., Jacobson T.M., Minter D.E., Cook P.F. (1993). Kinetic Mechanisms of the A and B Isozymes of O-Acetylserine Sulfhydrylase from Salmonella Typhimurium LT-2 Using the Natural and Alternate Reactants. Biochemistry.

[12] Toyomoto T., Ono K., Shiba T., Momitani K., Zhang T., Tsutsuki H., Ishikawa T., Hoso K., Hamada K., Rahman A. (2023). Alkyl Gallates Inhibit Serine O-Acetyltransferase in Bacteria and Enhance Susceptibility of Drug-Resistant Gram-Negative Bacteria to Antibiotics. Front. Microbiol..

[13] Verma D., Gupta S., Saxena R., Kaur P., Rachana R., Srivastava S., Gupta V. (2020). Allosteric Inhibition and Kinetic Characterization of Klebsiella Pneumoniae CysE: An Emerging Drug Target. Int. J. Biol. Macromol..

[14] Qin Y., Teng Y., Yang Y., Mao Z., Zhao S., Zhang N., Li X., Niu W. (2024). Advancements in Inhibitors of Crucial Enzymes in the Cysteine Biosynthetic Pathway: Serine Acetyltransferase and O-Acetylserine Sulfhydrylase. Chem. Biol. Drug Des..

[15] Ida T., Sawa T., Ihara H., Tsuchiya Y., Watanabe Y., Kumagai Y., Suematsu M., Motohashi H., Fujii S., Matsunaga T. (2014). Reactive Cysteine Persulfides and S-Polythiolation Regulate Oxidative Stress and Redox Signaling. Proc. Natl. Acad. Sci. USA.

[16] Sawa T., Motohashi H., Ihara H., Akaike T. (2020). Enzymatic Regulation and Biological Functions of Reactive Cysteine Persulfides and Polysulfides. Biomolecules.

[17] Barayeu U., Sawa T., Nishida M., Wei F.-Y., Motohashi H., Akaike T. (2023). Supersulfide Biology and Translational Medicine for Disease Control. Br. J. Pharmacol..

[18] Akaike T., Morita M., Ogata S., Yoshitake J., Jung M., Sekine H., Motohashi H., Barayeu U., Matsunaga T. (2024). New Aspects of Redox Signaling Mediated by Supersulfides in Health and Disease. Free Radic. Biol. Med..

[19] Ono K., Kitamura Y., Zhang T., Tsutsuki H., Rahman A., Ihara T., Akaike T., Sawa T. (2021). Cysteine Hydropersulfide Inactivates β-Lactam Antibiotics with Formation of Ring-Opened Carbothioic S-Acids in Bacteria. ACS Chem. Biol..

[20] Richard J.L., Debey M.C., Chermette R., Pier A.C., Hasegawa A., Lund A., Bratberg A.M., Padhye A.A., Connole M.D. (1994). Advances in Veterinary Mycology. J. Med. Vet. Mycol..

[21] Sutton P., Newcombe N.R., Waring P., Müllbacher A. (1994). In Vivo Immunosuppressive Activity of Gliotoxin, a Metabolite Produced by Human Pathogenic Fungi. Infect. Immun..

[22] Waring P., Beaver J. (1996). Gliotoxin and Related Epipolythiodioxopiperazines. Gen. Pharmacol. Vasc. Syst..

[23] Gardiner D.M., Waring P., Howlett B.J. (2005). The Epipolythiodioxopiperazine (ETP) Class of Fungal Toxins: Distribution, Mode of Action, Functions and Biosynthesis. Microbiology.

[24] Sasakura K., Hanaoka K., Shibuya N., Mikami Y., Kimura Y., Komatsu T., Ueno T., Terai T., Kimura H., Nagano T. (2011). Development of a Highly Selective Fluorescence Probe for Hydrogen Sulfide. J. Am. Chem. Soc..

[25] Hanaoka K., Sasakura K., Suwanai Y., Toma-Fukai S., Shimamoto K., Takano Y., Shibuya N., Terai T., Komatsu T., Ueno T. (2017). Discovery and Mechanistic Characterization of Selective Inhibitors of H2S-Producing Enzyme: 3-Mercaptopyruvate Sulfurtransferase (3MST) Targeting Active-Site Cysteine Persulfide. Sci. Rep..

[26] Kredich N.M., Foote L.J., Hulanicka M.D. (1975). Studies on the Mechanism of Inhibition of Salmonella Typhimurium by 1,2,4-Triazole. J. Biol. Chem..

[27] Scharf D.H., Brakhage A.A., Mukherjee P.K. (2016). Gliotoxin--Bane or Boon?. Environ. Microbiol..

[28] Smirnova G., Tyulenev A., Sutormina L., Kalashnikova T., Muzyka N., Ushakov V., Samoilova Z., Oktyabrsky O. (2024). Regulation of Cysteine Homeostasis and Its Effect on *Escherichia coli* Sensitivity to Ciprofloxacin in LB Medium. Int. J. Mol. Sci..

[29] Douafer H., Andrieu V., Phanstiel O., Brunel J.M. (2019). Antibiotic Adjuvants: Make Antibiotics Great Again!. J. Med. Chem..

[30] Li W., Tao Z., Zhou M., Jiang H., Wang L., Ji B., Zhao Y. (2024). Antibiotic Adjuvants against Multidrug-Resistant Gram-Negative Bacteria: Important Component of Future Antimicrobial Therapy. Microbiol. Res..

[31] Kumar V., Yasmeen N., Pandey A., Ahmad Chaudhary A., Alawam A.S., Ahmad Rudayni H., Islam A., Lakhawat S.S., Sharma P.K., Shahid M. (2023). Antibiotic Adjuvants: Synergistic Tool to Combat Multi-Drug Resistant Pathogens. Front. Cell. Infect. Microbiol..

[32] Claus M.T., Zocher G.E., Maier T.H.P., Schulz G.E. (2005). Structure of the O-Acetylserine Sulfhydrylase Isoenzyme CysM from *Escherichia coli*. Biochemistry.

[33] Spyrakis F., Singh R., Cozzini P., Campanini B., Salsi E., Felici P., Raboni S., Benedetti P., Cruciani G., Kellogg G.E. (2013). Isozyme-Specific Ligands for O-Acetylserine Sulfhydrylase, a Novel Antibiotic Target. PLoS ONE.

[34] Tai C.-H., Cook P.F. (2001). Pyridoxal 5’-Phosphate-Dependent α,β-Elimination Reactions:  Mechanism of O-Acetylserine Sulfhydrylase. Acc. Chem. Res..

[35] Zhou Y., Yu F., Chen M., Zhang Y., Qu Q., Wei Y., Xie C., Wu T., Liu Y., Zhang Z. (2021). Tylosin Inhibits Streptococcus Suis Biofilm Formation by Interacting with the O-Acetylserine (Thiol)-Lyase B CysM. Front. Vet. Sci..

[36] Brunner K., Maric S., Reshma R.S., Almqvist H., Seashore-Ludlow B., Gustavsson A.-L., Poyraz Ö., Yogeeswari P., Lundbäck T., Vallin M. (2016). Inhibitors of the Cysteine Synthase CysM with Antibacterial Potency against Dormant *Mycobacterium tuberculosis*. J. Med. Chem..

[37] Hurne A.M., Chai C.L.L., Waring P. (2000). Inactivation of Rabbit Muscle Creatine Kinase by Reversible Formation of an Internal Disulfide Bond Induced by the Fungal Toxin Gliotoxin. J. Biol. Chem..

[38] Waring P., Sjaarda A., Lin Q.H. (1995). Gliotoxin Inactivates Alcohol Dehydrogenase by Either Covalent Modification or Free Radical Damage Mediated by Redox Cycling. Biochem. Pharmacol..

[39] Bernardo P.H., Chai C.L.L., Deeble G.J., Liu X.-M., Waring P. (2001). Evidence for Gliotoxin–Glutathione Conjugate Adducts. Bioorg. Med. Chem. Lett..

[40] Salsi E., Bayden A.S., Spyrakis F., Amadasi A., Campanini B., Bettati S., Dodatko T., Cozzini P., Kellogg G.E., Cook P.F. (2010). Design of O-Acetylserine Sulfhydrylase Inhibitors by Mimicking Nature. J. Med. Chem..

[41] Pieroni M., Annunziato G., Beato C., Wouters R., Benoni R., Campanini B., Pertinhez T.A., Bettati S., Mozzarelli A., Costantino G. (2016). Rational Design, Synthesis, and Preliminary Structure-Activity Relationships of α-Substituted-2-Phenylcyclopropane Carboxylic Acids as Inhibitors of Salmonella Typhimurium O-Acetylserine Sulfhydrylase. J. Med. Chem..

[42] Annunziato G., Pieroni M., Benoni R., Campanini B., Pertinhez T.A., Pecchini C., Bruno A., Magalhães J., Bettati S., Franko N. (2016). Cyclopropane-1,2-Dicarboxylic Acids as New Tools for the Biophysical Investigation of O-Acetylserine Sulfhydrylases by Fluorimetric Methods and Saturation Transfer Difference (STD) NMR. J. Enzym. Inhib. Med. Chem..

[43] Magalhães J., Franko N., Annunziato G., Welch M., Dolan S.K., Bruno A., Mozzarelli A., Armao S., Jirgensons A., Pieroni M. (2018). Discovery of Novel Fragments Inhibiting O-Acetylserine Sulphhydrylase by Combining Scaffold Hopping and Ligand-Based Drug Design. J. Enzym. Inhib. Med. Chem..

[44] Marchetti M., De Angelis F.S., Annunziato G., Costantino G., Pieroni M., Ronda L., Mozzarelli A., Campanini B., Cannistraro S., Bizzarri A.R. (2021). A Competitive O-Acetylserine Sulfhydrylase Inhibitor Modulates the Formation of Cysteine Synthase Complex. Catalysts.

[45] Annunziato G., Spadini C., Marchetti M., Franko N., Pavone M., Iannarelli M., Bruno A., Pieroni M., Bettati S., Cabassi C.S. (2022). Inhibitors of O-Acetylserine Sulfhydrylase with a Cyclopropane-Carboxylic Acid Scaffold Are Effective Colistin Adjuvants in Gram Negative Bacteria. Pharmaceuticals.

[46] Poyraz Ö., Jeankumar V.U., Saxena S., Schnell R., Haraldsson M., Yogeeswari P., Sriram D., Schneider G. (2013). Structure-Guided Design of Novel Thiazolidine Inhibitors of O-Acetyl Serine Sulfhydrylase from *Mycobacterium tuberculosis*. J. Med. Chem..

[47] Brunner K., Steiner E.M., Reshma R.S., Sriram D., Schnell R., Schneider G. (2017). Profiling of in Vitro Activities of Urea-Based Inhibitors against Cysteine Synthases from *Mycobacterium tuberculosis*. Bioorg. Med. Chem. Lett..

[48] Franko N., Grammatoglou K., Campanini B., Costantino G., Jirgensons A., Mozzarelli A. (2018). Inhibition of O-Acetylserine Sulfhydrylase by Fluoroalanine Derivatives. J. Enzym. Inhib. Med. Chem..

[49] Wallace M.J., Dharuman S., Fernando D.M., Reeve S.M., Gee C.T., Yao J., Griffith E.C., Phelps G.A., Wright W.C., Elmore J.M. (2020). Discovery and Characterization of the Antimetabolite Action of Thioacetamide-Linked 1,2,3-Triazoles as Disruptors of Cysteine Biosynthesis in Gram-Negative Bacteria. ACS Infect. Dis..

[50] Chonoles Imlay K.R., Korshunov S., Imlay J.A. (2015). Physiological Roles and Adverse Effects of the Two Cystine Importers of *Escherichia coli*. J. Bacteriol..

[51] Ohtsu I., Kawano Y., Suzuki M., Morigasaki S., Saiki K., Yamazaki S., Nonaka G., Takagi H. (2015). Uptake of L-Cystine via an ABC Transporter Contributes Defense of Oxidative Stress in the L-Cystine Export-Dependent Manner in *Escherichia coli*. PLoS ONE.

[52] Sabrialabed S., Yang J.G., Yariv E., Ben-Tal N., Lewinson O. (2020). Substrate Recognition and ATPase Activity of the *E. coli* Cysteine/Cystine ABC Transporter YecSC-FliY. J. Biol. Chem..

[53] Ono K., Jung M., Zhang T., Tsutsuki H., Sezaki H., Ihara H., Wei F.-Y., Tomizawa K., Akaike T., Sawa T. (2017). Synthesis of L-Cysteine Derivatives Containing Stable Sulfur Isotopes and Application of This Synthesis to Reactive Sulfur Metabolome. Free Radic. Biol. Med..

