# Supplementary material for: Identification of Fungal Metabolite Gliotoxin as a Potent Inhibitor Against Bacterial O-Acetylserine Sulfhydrylase CysK and CysM
Source: Int J Mol Sci. 2025 Jan 27;26(3):1106. doi: 10.3390/ijms26031106 (PMC11818871; doi:10.3390/ijms26031106)
Supplement: Supplementary file 1 [file ijms-26-01106-s001.zip › ijms-3365087-supplementary.pdf]

Supporting information for

Identification of fungal metabolite gliotoxin as a potent inhibitor against bacterial *O*-acetylserine sulfhydrylase CysK and CysM

Azizur Rahman[1,#], Katsuhiko Ono[1,#], Touya Toyomoto[1], Kenjiro Hanaoka[2], and Tomohiro Sawa[1,*]

[1]Department of Microbiology, Graduate School of Medical Sciences, Kumamoto University
[2]Division of Analytical Chemistry for Drug Discovery, Faculty of Pharmacy, Graduate School of Pharmaceutical Sciences, Keio University

[#]These authors equally contributed to this work.
[*]correspondence: sawat@kumamoto-u.ac.jp

Contents:

Supplementary Figure S1. H_2S consumption by CysM determined by HSip-1 assay.
Supplementary Figure S2. Representative data for high-throughput screening of OASS inhibitors.
Supplementary Figure S3. Results of the first and second screening of OASS inhibitors.
Supplementary Figure S4. Bioassay of OASS inhibitors.
Supplementary Figure S5. Effects of gliotoxin on HSip-1-derived fluorescent intensity in the presence of H_2S.
Supplementary Figure S6. Inhibition of CysK (A) and CysM (B) by gliotoxin as determined by cysteine production.
Supplementary Figure S7. Effects of 2-oxo-1,3-dithiole-4,5-dicarboxamide on the growth of *S.* Typhimurium LT2.

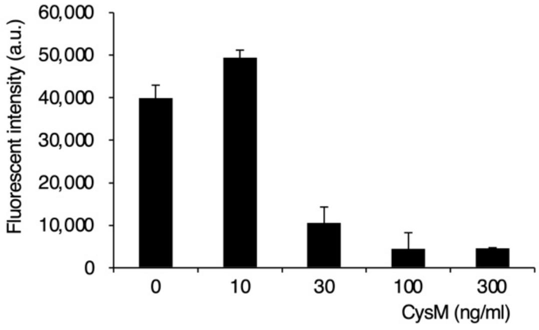

Supplementary Figure S1. H_2S consumption by CysM determined by HSip-1 assay. HSip-1 derived fluorescent intensities were measured in reaction mixtures containing 100 μM OAS, 100 μM NaHS, 1 μM HSip-1 with indicated concentrations of CysM.

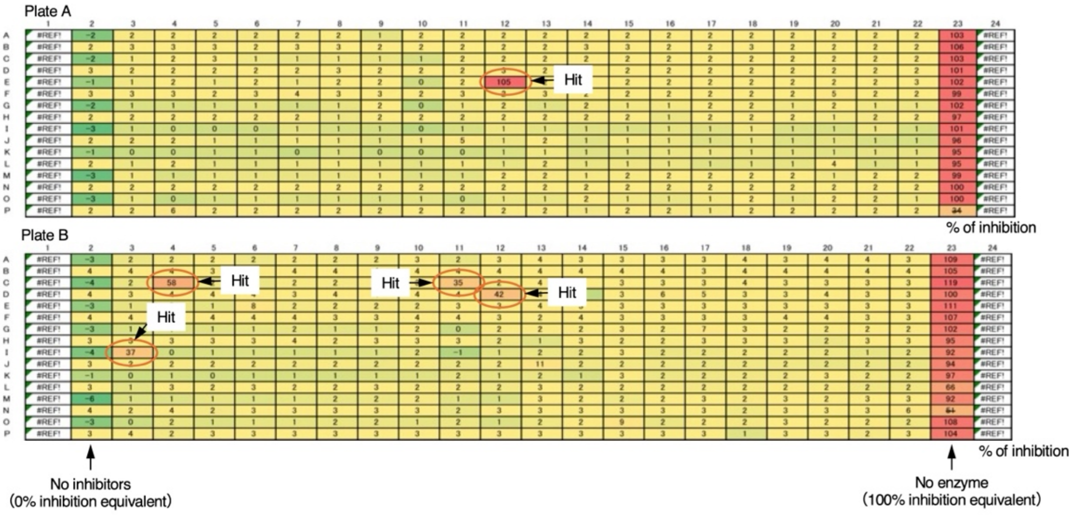

Supplementary Figure S2. Representative data for high-throughput screening of OASS inhibitors.

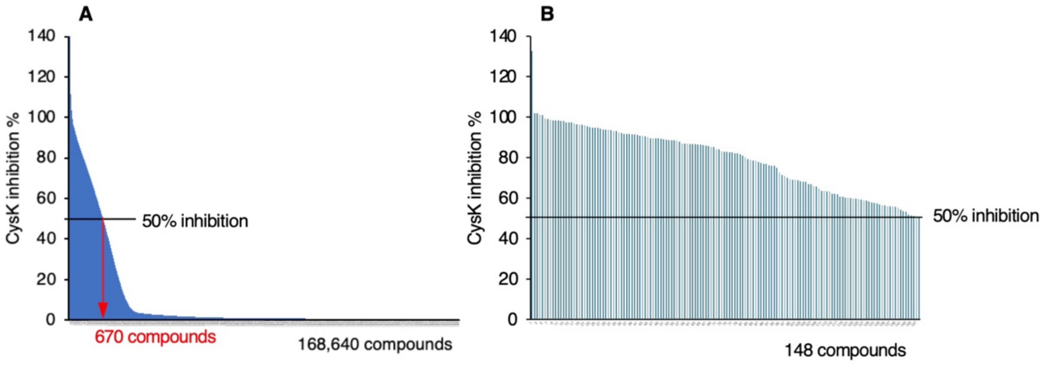

Supplementary Figure S3. Results of the first and second screening of OASS inhibitors. Total 168,640 compounds were analyzed their effects on CysK enzyme reactions (A). The fifty % inhibition was used for cut-off value to identify 670 compounds as candidates for the second screening. In the second screening (B), 148 compounds were finally selected for bioassay.

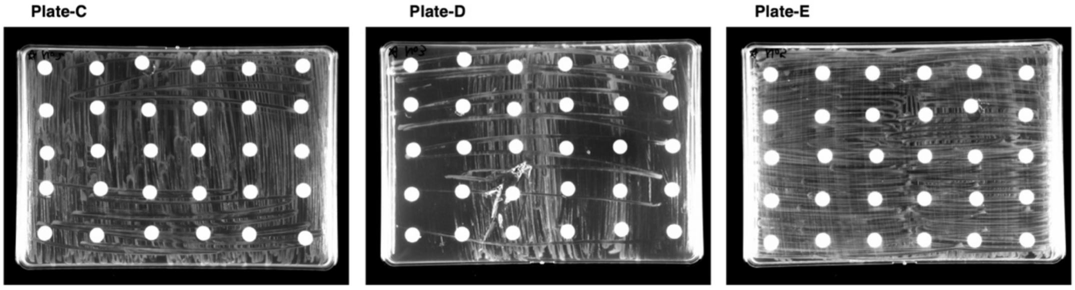

Supplementary Figure S4. Bioassay of OASS inhibitors. After enzyme-based screening, 148 compounds were subjected for bioassay. Data for Plate-A and Plate-B were shown in Figure 7. As shown in this figure, no positive zone of inhibition was determined for the compounds examined.

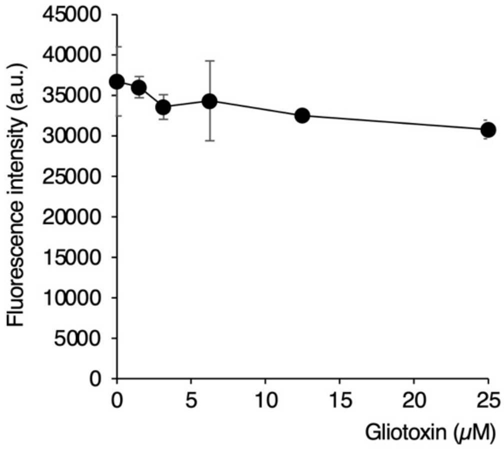

Supplementary Figure S5. Effects of gliotoxin on HSip-1-derived fluorescent intensity in the presence of H_2S. NaHS (300 μM) was incubated with indicated concentrations of gliotoxin at room temperature for 1 h, followed by further incubating with 1 μM HSip-1 for 3 h. Fluorescence intensities of the reaction mixtures were measured with excitation at 485 nm and with emission at 535 nm. Data are means ±SD (n=3).

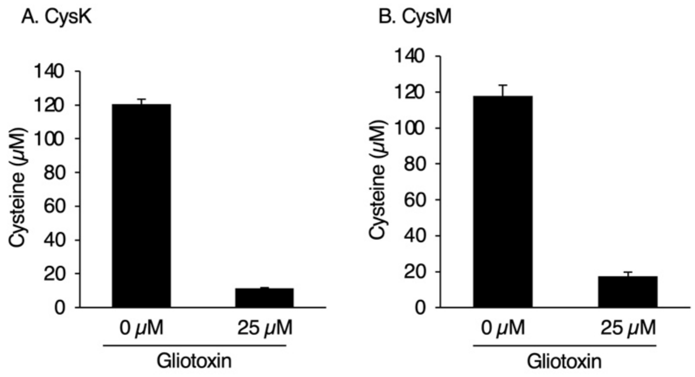

Supplementary Figure S6. Inhibition of CysK (A) and CysM (B) by gliotoxin as determined by cysteine production. The reaction mixtures contained 25 ng/ml enzymes, 300 μM NaHS, 1 mM OAS and 25 μM of gliotoxin. Data are means ±SD (n=3).

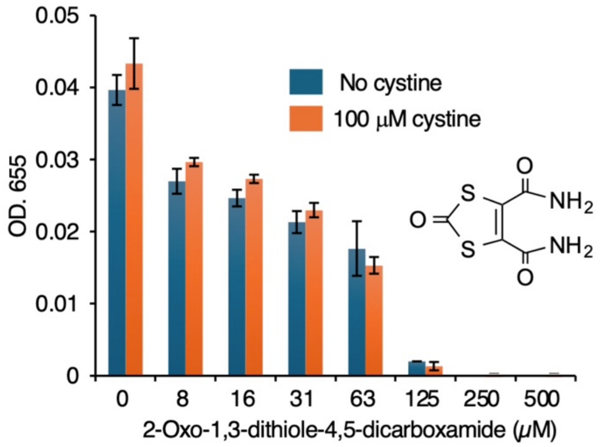

Supplementary Figure S7. Effects of 2-oxo-1,3-dithiole-4,5-dicarboxamide on the growth of *S.* Typhimurium. *S.* Typhimurium were cultured in M9 medium without or with 100 µM cystine containing indicated concentrations of the reagent for 24 h. Bacterial growth was determined by measuring turbidity. Data are means ± SD (n = 3). Chemical structure of 2-oxo-1,3-dithiole-4,5-dicarboxamide is shown in this figure.
